# Clinical application of endoscopic surgery using a gasless unilateral transaxillary approach in the treatment of primary hyperparathyroidism

**DOI:** 10.3389/fsurg.2022.962463

**Published:** 2022-09-22

**Authors:** Wan-Chen Zhang, Dong-Ning Lu, Jia-Jie Xu, Hai-Wei Guo, Ming-Hua Ge, Chuan-Ming Zheng

**Affiliations:** ^1^ Second Clinical Medical College, Zhejiang Chinese Medical University, Hangzhou, China; ^2^ Otolaryngology / Head and Neck Center, Cancer Center, Department of Head and Neck Surgery, Zhejiang Provincial People's Hospital (Affliated People's Hospital, Hangzhou Medical College), Hangzhou, China; ^3^ Key Laboratory of Endocrine Gland Diseases of Zhejiang Province, Hangzhou, China

**Keywords:** axillary approach, minimally invasive surgery, primary hyperparathyroidism, endoscopic, parathyroidectomy

## Abstract

**Objectives:**

To investigate the safety and feasibility of gasless axillary parathyroid surgery in the treatment of primary hyperparathyroidism.

**Methods:**

A total of 12 patients who received gasless axillary parathyroidectomy (endoscope group) and 14 patients who received traditional open parathyroidectomy (open group) from January 2019 to April 2022 were screened and included. The differences in baseline characteristics, surgical efficiency, incidence rate of complications, changes in biochemical indicators, and incision satisfaction between the two groups were analyzed and compared.

**Results:**

The proportion of young patients was higher in the endoscopic group than in the open group, and the difference was statistically significant [(41.33 ± 13.65) years vs. (58.00 ± 9.44) years, *P* < 0.01]. The differences in operation time, intra-operative blood loss, post-operative drainage volume, hospital stay, and surgical efficiency between the two groups yielded no statistical significance (*P* > 0.05). Patients in the open group had more significant neck pain 3 days after surgery (*P* = 0.046), but the degree of pain 3 months after surgery was the same in the 2 groups (*P* = 0.432). Evaluation of post-operative mature stage scar and incision satisfaction regarding aesthetics in the endoscope group were significantly superior to that in the open group [(1.92 ± 0.92) points vs. (0.92 ± 1.00) points, *P* = 0.017 and (1.57 ± 0.51) points vs. (1.00 ± 0.013) points, *P* = 0.013, respectively]. No statistical significance was found in terms of incidence rate of post-operative fever (*P* > 0.05). No temporary recurrent laryngeal nerve injury, post-operative bleeding, incision hematoma infection, or other complications were observed. Comparing the two groups, the extent of the level decrease of PTH was similar to that of serum calcium and phosphorus (*P* < 0.05), where most patients experienced transient hypocalcemia after operation yielding no significant difference in incidence (*P* = 0.225). During a follow-up period of 3 to 36 months, a total of 1 patient in the open group experienced recurrence at 10 months after surgery and was treated non-surgically.

**Conclusion:**

Gasless axillary approach to parathyroid surgery for primary hyperparathyroidism possesses good safety and patient satisfaction in terms of aesthetics.

## Introduction

Primary hyperparathyroidism (PHPT) is one of the more common endocrine diseases, characterized by high serum calcium and parathyroid hormone levels. It is mainly caused by single benign parathyroid tumors that account for 80%–85% of cases, while multiple adenomas account for about 10%–15%, and parathyroid carcinoma less than 1% (1). Currently, surgical resection is the most effective treatment to relieve clinical symptoms and reduce the incidence of complications (2).

Traditional open surgery leaves an obvious cervical incision scar, which damages the aesthetics of the neck and the function of the anterior cervical region (such as swallowing and sensation). Manifesting most commonly in young and middle-aged women, this may lead to adverse psychological and physiological effects (3).

In the past two decades, with the development of preoperative imaging technology and endoscopic surgery, thyroid and parathyroid surgery have been widely popularized and applied, opening a variety of approaches to external cervical thyroid and parathyroid surgery. In 2017, Ge Minghua et al. carried out and modified endoscopic thyroidectomy using a gasless unilateral axillary approach (GUA), which gained roots in China, and thereafter became a well-rounded GUA system that has serviced many regions to great effect (4). By successfully applying such a method in parathyroid tumor resection surgery where all patients were able to achieve good therapeutic results, we have further pushed the frontier of GUA's potential applications.

At present, there are no controlled studies that compare traditional open parathyroidectomy (open group) and GUA parathyroidectomy (endoscopic group). This study will summarize and analyze the diagnosis and treatment, clinicopathological data, and follow-up data of patients with PHPT at our center, to assess the merits of GUA in terms of safety and feasibility in parathyroid surgery to treat PHPT patients.

## Materials and methods

### General information

Clinical data of patients treated at the Department of Head and Neck Surgery, Zhejiang Provincial People's Hospital from January 2019 to April 2022 were collected. To reduce the bias introduced by the sample characteristics, we used bivariate logistic regression for propensity score matching whenever possible. Finally, 12 received gasless axillary parathyroidectomy (endoscope group) and 14 matched patients who received traditional open parathyroidectomy (open group) were selected. All operations were performed by the same experienced surgeon, and the type of operation was selected by the patients after being wholly informed. There were no instances of need for patients in the endoscope group to resort to open surgery in our cohort. All patients and their families signed the informed consent form for surgery.

Inclusion criteria: Lesion sites for all patients were able to be accurately located using color Doppler ultrasound, ^99^Tcm-MIBI, or CT scan before surgery. All were clinically diagnosed with PHPT based on serological tests for parathyroid hormone and serum calcium levels along with single parathyroid adenoma through imaging. No patient had previous history of neck trauma, neck radiation, or surgery.

Exclusion Criteria: Patients with secondary hyperparathyroidism, severe comorbidities, history of parathyroidectomy, are suspected to be with polyglandular disease, or are lost to follow-up.

### Surgical methods (parathyroid adenoma in the left upper pole)

#### Surgical approach of endoscope group

**Figure 1:**
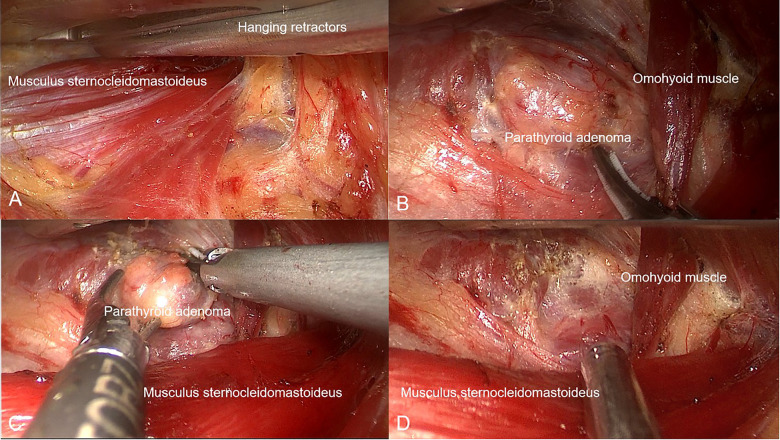
Unilateral gasless axillary access parathyroid surgery step (**A**) separate the sternal head of the sternocleidomastoid muscle from the clavicular head. (**B**) Exploration was performed to expose the parathyroid tumor in the left upper pole. (**C**) Complete excision of this parathyroid tumor. (**D**) Full display of resected surgical cavity.

After general anesthesia, the patient was placed in the supine position, with shoulders padded, and head slightly tilting to the unaffected side. Routine cervicothoracic, axillary disinfection, and draping were performed. A naturally wrinkled skin incision about 4 cm in length was made in the left axilla. The skin and subcutaneous tissue were incised and separated along the surface of the subcutaneous pectoralis major muscle. Instruments are then inserted utilizing the natural space between the sternal head of the sternocleidomastoid muscle and the head of the clavicle. Muscles are then suspended using devices assembled in the GUA space (Figure 1A). The natural space between the thyroid gland and the anterior cervical strap muscle was then expanded. The retractor was fixed after pulling the anterior cervical strap muscle to expose the lateral rear of the left thyroid gland (Figure 1B). The recurrent laryngeal nerve was sought, isolated, and protected in the tracheoesophageal groove. The middle thyroid vein was transected. The parathyroid tumor behind the left upper pole of the thyroid gland was then identified (Figure 1C). When the tumor was removed from the body for 10 min, the intra-operative parathyroid hormone (IOPTH) value was detected. Intra-operative frozen section was acquired, exact hemostasis was performed, and the surgical cavity was irrigated (Figure 1D). Upon pathologically confirming the frozen specimen to be parathyroid adenoma, the surgical cavity was closed and a negative pressure drainage tube was placed (Supplementary video 1).

#### Surgical approach of open group

After general anesthesia, the patient was placed in a supine head-up extension position. An anterior low cervical incision of 4 cm–6 cm incision in size was made along the dermatoglyphic line 2 cm above the manubrium sterni, and the skin, subcutaneous fascia, and platysma muscle were incised layer by layer. The anterior cervical flap was freed, anterior cervical muscle group opened along the linea alba, and the tissue around the left thyroid gland separated. The left upper pole parathyroid tumor was then isolated and exposed, the thyroid gland pulled to the unaffected side, and the recurrent laryngeal nerve isolated and protected. The upper pole thyroid vessels and the ascending branch of the inferior thyroid artery were transected and ligated, then the left upper pole dorsal parathyroid tumor was completely removed for intra-operative frozen section diagnosis. Hemostasis and washing ensued, then a negative pressure drainage tube was placed.

### Outcome measurements

The clinicopathological data of the patients were collected, and a database was established to record the general data of the patients such as gender, age, location and size of the parathyroid tumor, operation time, intra-operative blood loss, post-operative drainage volume, hospital stay, and follow-up time, as well as parathyroid hormone, serum calcium, and serum phosphorus levels before surgery, 10 min after gland excision, 1 day after surgery and 1 month after surgery. Patients were considered to have been surgically treated successfully if their blood calcium levels and post-operative parathyroid hormone levels returned to normal for those whose levels were abnormally high pre-operation. For patients whose pre-operative blood calcium levels were normal yet had high levels of PTH, success of treatment were based on whether parathyroid hormone levels returned to normal. Recurrence was defined as: detected hypercalcemia in 6 months and above follow-up after successful surgical treatment of parathyroid glands. Adverse events and complications included intra-operative exploration failure, recurrent laryngeal nerve injury, wound infection, incisional hematoma, permanent hypoparathyroidism, readmission, and death. In addition, we made supplementary post-operative recovery assessments from an aesthetic point of view using the SCAR scale and also post-operative VAS pain score.

### Statistical analysis

All data were analyzed using SPSS 25.0 software, and the data of the endoscope and open groups were compared using the two-sample *t*-test, Wilcoxon rank-sum test, chi-square test, or Fisher's exact test. Measurement data results were expressed as mean ± standard deviation (*x¯* ± *s*), enumeration data were expressed as number (%), hazard ratio (HR) was reported with a 95% confidence interval (95% CI), and *P* < 0.05 was considered statistically significant.

## Results

### Baseline characteristics

A total of 26 patients were enrolled, including 14 in the open group and 12 in the endoscopic group. The age of the endoscopic group was (41.33 ± 13.65) years, which was younger than that of the open group (58.00 ± 9.44) years, and the difference was statistically significant (*P* < 0.01). The two groups were comparable in terms of gender, longest diameter of the lesion, location of the lesion, and follow-up time, as shown in Table 1. Post-operative routine pathological report returned parathyroid adenoma in all cases.

**Table 1:** Characteristics of patients and tumor baseline in two groups.

	Open group (*n* = 14)	Endoscope group (*n* = 12)	*P*-value
Age (years)	58.00 ± 9.44	41.33 ± 13.65	0.001
Sex			0.401
Male	3 (21.43)	5 (41.67)	
Female	11 (78.57)	7 (58.33)	
BMI (kg/m^2^)	23.33 ± 3.31	22.83 ± 3.04	0.693
Tumor diameter (mm)	20.07 ± 7.08	16.08 ± 6.22	0.143
Tumor location			
Left	5 (35.71)	5 (41.67)	0.536
Right	9 (64.29)	7 (58.33)	
Upper pole	4 (28.57)	5 (41.67)	0.387
Lower pole	10 (71.43)	7 (58.33)	
Follow-up time (day)	22.86 ± 13.93	18.75 ± 10.53	0.589

### Surgical assessment

As shown in Table 2, the differences in operation time, intra-operative blood loss, post-operative hospital stay, and post-operative drainage volume between the open and endoscope group were non-significant (*P* > 0.05). There were 11 cases of post-operative numbness in the hand and foot for the open group and 12 cases for the endoscope group. The symptoms were transient, and no statistical difference was found between the two groups (*P* = 0.225). There were cases of fever post-operation in the two groups, but the differences were non-significant (*P* = 0.671). None had wound infection, incision edema, skin ecchymosis, or recurrent laryngeal nerve injury. The VAS pain score at 3 days after operation, along with proliferative stage SCAR scale assessment and incision satisfaction were all significantly superior in the endoscope group when compared to the open group (*P* < 0.05). There were no significant differences in VAS pain score at 3 months after operation and mature stage SCAR scale assessment between the two groups (*P* > 0.05).

**Table 2:** Comparison of intraoperative and postoperative conditions.

	Open group (*n* = 14)	Endoscope group (*n* = 12)	*P*-value
Operation time (min)	61.79 ± 26.36	52.92 ± 13.56	0.469
Intraoperative blood loss (ml)	10.43 ± 1.60	10.25 ± 1.29	0.860
Postoperative hospital stay (day)	4.64 ± 1.82	4.42 ± 1.16	0.715
Postoperative drainage (ml)	90.77 ± 61.92	121.73 ± 62.18	0.236
**Complications**			
Voice hoarse	0 (0.00)	0 (0.00)	–
Limbs numb	11 (78.57)	12 (100)	0.225
Fever	7 (50.00)	5 (41.67)	0.671
Infection	0 (0.00)	0 (0.00)	–
Skin ecchymosis	0 (0.00)	0 (0.00)	–
Recurrence	1 (0.07)	0 (0.00)	0.538
**Postoperative neck VAS score**			
3rd days after	1.64 ± 0.50	1.00 ± 0.95	0.046
3 months after	0.14 ± 0.36	0.33 ± 0.49	0.432
**Postoperative chest VAS score**			
3rd days after	–	1.08 ± 0.51	–
3 months after	–	0.67 ± 0.78	–
**Scar scale assessment**			
Proliferative stage	2.57 ± 1.16	2.08 ± 0.90	0.248
Mature stage	1.92 ± 0.92	0.92 ± 1.00	0.017
Incision satisfaction	1.57 ± 0.51	1.00 ± 0.00	0.013

### Therapeutic feasibility

No heterogeneity was found for preoperative PTH, serum calcium, serum phosphorus, and calcium-phosphorus product between the two groups, suggesting they had baseline comparability. PTH concentration decreased by more than 50%, reaching normal ranges 10 min after gland excision in both groups, and there was no significant difference in PTH, serum calcium, hemophosphate, and calcium-phosphorus product 1 day after the operation and 1 month after operation (*P* > 0.05) in both groups. During follow-up, one patient in the open group experienced hypercalcemia and PTH increase 10 months after operation, and was considered to be a case of recurrence. There were no cases of post-operative recurrence in the endoscope group (Table 3).

**Table 3:** Changes of PTH, serum calcium and serum phosphorus in all patients before and after surgery.

	Preoperative			10 min after gland excision			1 day after operation			1 month after operation		
	Open group (*n* = 14)	Endoscope group (*n* = 12)	*P*-value	Open group (*n* = 14)	Endoscope group (*n* = 12)	*P*-value	Open group (*n* = 14)	Endoscope group (*n* = 12)	*P*-value	Open group (*n* = 14)	Endoscope group (*n* = 12)	*P*-value
PTH (ng/L)	346.81 ± 435.42	225.44 ± 158.79	0.372	42.07 ± 10.67	40.16 ± 15.64	0.715	23.90 ± 15.53	28.27 ± 24.46	0.787	104.38 ± 98.75	50.53 ± 43.24	0.172
Serum calcium (mmol/L)	2.76 ± 0.24	2.72 ± 0.31	0.757	–	–	–	2.24 ± 0.14	2.22 ± 0.17	0.657	2.25 ± 0.13	2.31 ± 0.09	0.374
Serum phosphorus (mmol/L)	0.95 ± 0.16	0.82 ± 0.15	0.056	–	–	–	1.14 ± 0.42	0.97 ± 0.21	0.369	1.23 ± 0.21	1.20 ± 0.20	0.771
Calcium-phosphorus product	2.60 ± 0.39	2.17 ± 0.40	0.014	–	–	–	1.17 ± 1.48	2.17 ± 0.49	0.102	2.78 ± 0.60	2.79 ± 0.56	0.976

Reference value: PTH: 11.0–67.0 pg/ml, serum calcium: 2.11–2.52 mmol/L, serum phosphorus: 0.85–1.51 mmol/L.

## Discussion

PHPT arises from primary parathyroid lesions and may present with hypercalcemia, in forms of unexplained urolithiasis, hematuria, urinary tract infection, bone pain, pathological fractures, or intractable peptic ulcer, as well as muscle weakness or abnormal psychiatric symptoms. Single parathyroid adenoma is the most common pathological subtype, and 95% of patients with PHPT were able to have their hypercalcemia and related complications effectively reduced through surgery (2). Diagnosis and localization are key to preoperative evaluation. With the rapid development of imaging technology, the sensitivity of preoperative parathyroid localization can reach 92.0% (5), which is helpful for the successful implementation of surgery.

Prior to the 1990s, open neck exploration with cervical incision was mostly used for PHPT to expose all parathyroid glands and remove hyperfunctioning tissues. According to the 2016 American Association of Endocrine Surgeons Guidelines for the Management of Primary Hyperparathyroidism, endoscopic parathyroid tumor resection can be applied to patients with single parathyroid adenoma that have been diagnosed clinically or radiologically (6). Therefore, an increasing number of patients and doctors shied away from cervical incision and opted to use breast, oral cavity, and axillary approaches to lessen trauma and achieve faster recovery. In 2002, Ikeda et al. (7) first reported CO2-inflated axillary access thyroid surgery for the treatment of thyroid follicular tumors, Graves' disease, and thyroid micropapillary carcinoma. In 2003, Chung et al. (8) first carried out “gasless endoscopic thyroidectomy via an axillary approach”. In 2005, it was further improved by Tae et al. (9) becoming “a gasless unilateral axillo-breast or axillary approach endoscopic thyroidectomy”. Our team introduced the gasless axillary thyroid surgery into China for the first time in 2017, and introduced further improvements and innovations, including the innovative axilla cosmetic incision, selection of first or second axillary fold line, along with concealment and cosmetic improvements of the incision. We also redesigned the assembly equipment for gasless endoscopic thyroid surgery that has been registered and protected by intellectual property rights under “Ge / Zheng's seven-step method” (10). These were key in boosting convenience during operation and also shortened the learning curve for surgeons and assistants (11). Given the maturation of endoscopic surgery via the gasless axillary approach, we believe it is time to apply it to the resection of parathyroid tumors located laterally so unique anatomical advantages along this pathway could be adequately exploited, removing the need to dig behind the thyroid. Given the angle of attack, the same approach may also see great utility in addressing other tumors located to the lateral side of the neck.

In terms of the baseline characteristics of the patients included in the study, due to the high cosmetic requirements for surgery in young patients, it was difficult to avoid the difference in the age factors given the patient-selected incision approach. To minimize bias, we excluded patients aged >70 years or with other chronic diseases to minimize the impact of factors such as age. There were no differences in other indicators such as gender, BMI, tumor size, tumor location, serum calcium, phosphorus, and PTH.

Traditionally, endoscopic surgery *via* the non-cervical approach requires more surgical time than the cervical approach because it requires lacunae, as previously demonstrated in studies of thyroid surgery (11, 12). However, there was no difference in operation time between endoscope group and open group in this study, and the average operation time of endoscope group in this study was shorter than previous transaxillary robot-assisted parathyroidectomy [(52.92 ± 13.56 min) vs. (116.00 ± 53.00 min)] (13). This may be due to the intra-operative convenience brought forth through our modifications where after the operation space is established, the parathyroid adenoma located on the posterior side of the thyroid gland is easily and fully exposed in the field of view, presenting the inferior parathyroid adenoma for immediate complete resection. In addition, “three-step cavity construction method” and “natural gap cavity construction concept” were key changes made to accelerate cavity construction, fully exploiting the natural cavity of the neck in tandem with the auxiliary devices such as special hanging retractors. Presenting a clear and continuous smoke-free surgical space for the surgeon further eliminates the time needed to wipe lens due to smoke interference.

This study suggested that the post-operative drainage volume in the endoscopic group was not different from that in the open group. This disagrees with a previous comparative study results of thyroidectomy (14) and could be due to the following reasons: first, the resection of axillary parathyroid glands does not require additional free anterior cervical flap, and the expansion of the surgical trauma is limited; second, surgeons familiar with the operation and can build a cavity with the help of the natural space of the cervical muscles to minimize the damage caused by axillary to cervical cavity building; third, the axillary approach performs parathyroid tumor resection from the lateral approach without damaging the thyroid gland and lymphoid tissue.

There were no significant differences in post-operative hospital stay and intra-operative blood loss between the two groups in the study, suggesting that GUA parathyroidectomy will not bring additional surgical trauma to the patients.

In terms of surgical complications, patients in the endoscope group and open group had a higher incidence rate of post-operative transient numbness in hands and feet, which was also reported in previous literature (15). This could be explained by uncompensated inhibition of remaining parathyroid secretion after removal of the hyperfunctioning adenoma, leading to temporary hypoparathyroidism. Therefore, for patients with post-operative numbness of hands and feet or serum calcium lower than normal level (<0.25), we prescribed short-term routine calcium supplement and calcitriol supplement when necessary. In addition, 7 patients in open group and 5 patients in endoscope group had post-operative transient fever (axillary temperature 37.3–38.0 °C), which was caused by surgical stress. Body temperatures all returned to normal at discharge. No other adverse complications were observed. Statistical analysis showed that there were no significant differences in the incidence of post-operative complications between the two groups. This study did not find any incidence of acute complications such as transient recurrent laryngeal nerve injury, incisional hemorrhage and hematoma, perhaps thanks to the extensive surgical experience of the surgical team.

**Figure 2:**
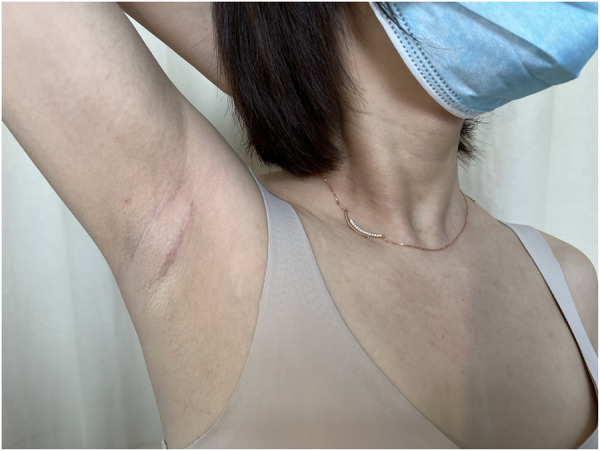
Post-operative incision recovery.

In the evaluation of post-operative recovery of patients, the open group had more significant neck pain 3 days after operation compared to the endoscope group due to the incision in front of the neck. There was no difference in neck pain 3 months after operation between the two groups. Due to cavity construction in the axillary approach, there may be experiences of transient precordial discomfort which will disappear within 3 months after surgery for both approaches. For scaring, we used the SCAR scale to perform a more comprehensive assessment from many aspects such as the patient and physician viewed scar appearance and sensation. There were no significant differences in scar score during the proliferative stage between the endoscopic group and the open group, but the score in the endoscopic group was significantly lower than that in the open group during the mature stage. We believe that the reason for this difference is that the natural fold of the axilla well masks the presence of the scar, and there is no significant tension in the axillary incision, making it less likely to form scar tissues (Figure 2). In terms of cosmetic satisfaction, the advantages of axillary scar were more obvious. All patients participating in axillary parathyroidectomy are very satisfied with the resulting aesthetics, and this could contribute to the recovery of their physical and mental health, along with self-confidence.

During post-operative follow-up, a patient in the open group experienced hypercalcemia and abnormal PTH 10 months after operation. Considering that compared to first-time surgery, the healing rate of repeated surgeries decreases by 2.0%–18.0% while carrying higher risks (16), we recommended close monitoring, evaluation of biochemical indicators and drug treatment. There were no cases of recurrence in the endoscope group.

This study preliminarily demonstrated the safety and feasibility of the gasless axillary approach to parathyroid tumor resection, yielding good post-operative results in terms of prognosis and aesthetics. However, there are some limitations in this study due to the sample size. A multicenter study with a larger sample size should be conducted in the future to further demonstrate the generalizability of this study in patients of all ages and regions.

## Summary

In this article, we introduced the application of non-pneumatic axillary approach surgery in the surgical process of parathyroid adenoma in detail, demonstrating its advantages in terms of safety, convenience, and aesthetics through comparative analysis. This provides an additional reference for the surgical treatment of parathyroid gland tumors, and for the diagnosis and treatment of tumors located on the lateral side of the neck.

## Data Availability

The original contributions presented in the study are included in the article/Supplementary Material, further inquiries can be directed to the corresponding author/s.
